# Successful tocilizumab treatment for rapidly progressive interstitial lung disease with anti-MDA5-positive juvenile dermatomyositis: a case report and literature review

**DOI:** 10.3389/fped.2024.1497168

**Published:** 2024-11-27

**Authors:** Lingzhi Qiu, Xiaoyan Shao, Le Ma, Zhidan Fan, Haiguo Yu

**Affiliations:** Department of Rheumatology and Immunology, Children’s Hospital of Nanjing Medical University, Nanjing, China

**Keywords:** anti-MDA5, juvenile dermatomyositis, rapidly progressive interstitial lung disease, tocilizumab, plasma exchange

## Abstract

**Background:**

Currently, no established integrated treatment regimen exists for anti-melanoma differentiation-associated gene 5 (anti-MDA5)-positive juvenile dermatomyositis (JDM) complicated by rapidly progressive interstitial lung disease (RP-ILD). We present a case of refractory anti-MDA5-positive JDM with RP-ILD that was successfully treated using a combination of tocilizumab and plasma exchange, along with a review of the relevant literature.

**Methods:**

A literature review was conducted to gain insights into the clinical features and treatment strategies for managing refractory anti-MDA5-positive JDM complicated by RP-ILD.

**Results:**

We report a case of successful management of anti-MDA5-positive JDM complicated by RP-ILD using a combination of immunosuppressive agents, plasma exchange, and tocilizumab.

**Conclusion:**

Tocilizumab may serve as an effective adjunctive treatment option for patients with refractory anti-MDA5-positive JDM complicated by RP-ILD who do not respond to conventional intensive immunosuppressive therapies.

## 1 Introduction

Juvenile dermatomyositis (JDM) is a rare childhood-onset systemic inflammatory disease, characterized by typical cutaneous lesions, such as Gottron's papules and heliotrope rash, as well as proximal muscle weakness. Anti-melanoma differentiation-associated gene 5 (anti-MDA5)-positive JDM often coexists with rapidly progressive interstitial lung disease (RP-ILD), which is associated with high mortality and can be life-threatening. Currently, there exists no consensus on the optimal treatment plan for patients with anti-MDA5-positive JDM complicated by RP-ILD. Herein, we present a case of a 3-year-old girl patient with anti-MDA5-positive JDM complicated by RP-ILD, whose lung condition continued to worsen on imaging despite receiving multiple immunosuppressive intensive therapies, including intravenous immunoglobulin (IVIG), glucocorticoid, tofacitinib, tacrolimus, and cyclophosphamide. Rescue treatments with plasma exchange and tocilizumab were subsequently initiated. Significant pulmonary improvement was observed, as evidenced by lung imaging and resolution of hypoxemia after three sessions of plasma exchange and tocilizumab therapy. Notably, the most substantial improvement was observed after tocilizumab therapy. To the best of our knowledge, this case represents the first reported instance of successful tocilizumab use in an anti-MDA5-positive JDM patient complicated by RP-ILD. Furthermore, tocilizumab demonstrated both efficacy and safety in our case, providing a new idea for the future treatment of refractory anti-MDA5-positive JDM complicated by RP-ILD.

## 2 Case presentation

**Figure 1:**
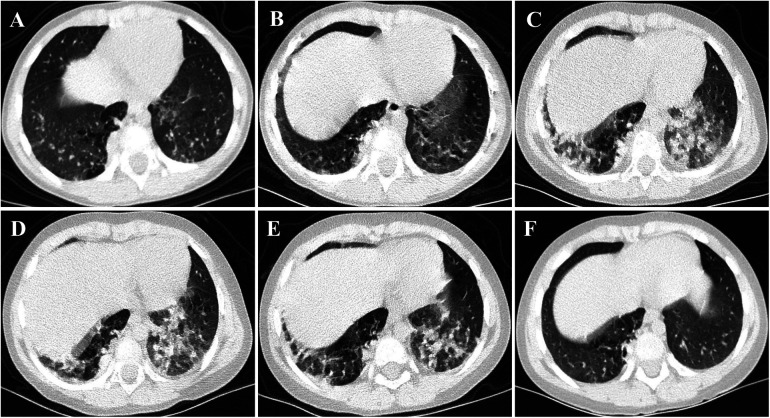
CT images of the case. (**A,B**) Flocculent shadows were observed in both lungs, accompanied by consolidation in the right lung at disease onset. After the initial treatment, these lesions were slightly absorbed. (**C,D**) Multiple patchy subpleural exudates and interstitial changes were noted in both lungs, with worsening consolidation in the right lung compared to the previous state. (**E,F**) Following combination therapy, notably with tocilizumab, substantial improvement was observed in both subpleural exudation and consolidation.

**Figure 2:**
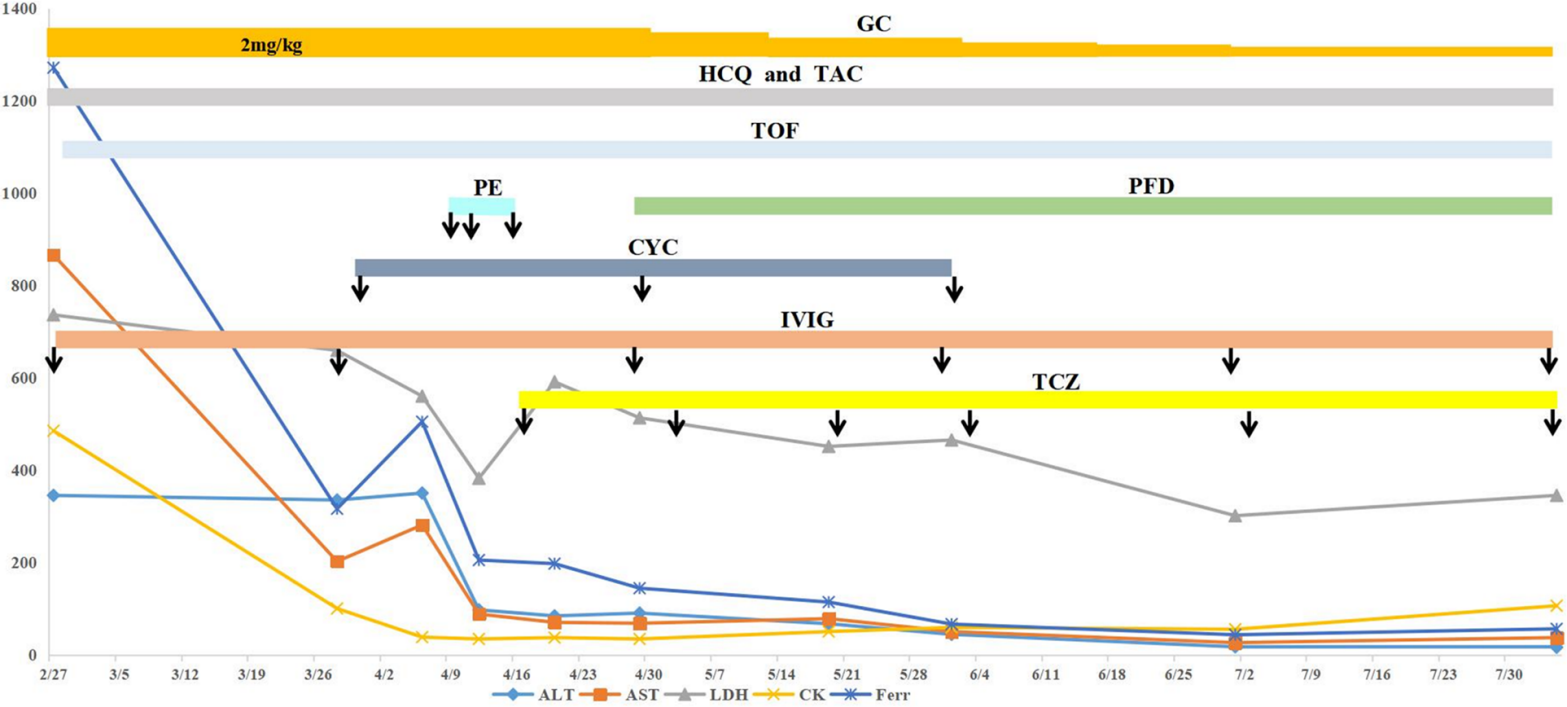
Clinical course of the case. The patient received combination therapy, including oral hydroxychloroquine, tofacitinib, tacrolimus, glucocorticoid (tapered off gradually), IVIG (2 g/kg/dose at a 1-month interval), cyclophosphamide (500–700 mg/m^2^/dose, once a month for three sessions), and regular tocilizumab. The arrow indicates the administration of the marked drugs. After treatment, ALT, AST, LDH, CK, and Ferr levels were all improved to different degrees. ALT, alanine transaminase; AST, aspartate aminotransferase; LDH, lactic dehydrogenase; CK, creatine kinase; Ferr, ferritin; HCQ, hydroxychloroquine; GC, glucocorticoid; TAC, tacrolimus; TOF, tofacitinib; PE, plasma exchange; PFD, pirfenidone; CYC, cyclophosphamide; IVIG, intravenous immunoglobulin; TCZ, tocilizumab.

A 3-year-old girl was admitted to the hospital due to recurrent rashes for 3 months and limb weakness for 2 months. Physical examination revealed multiple red maculopapules on her face, bilateral elbows, interphalangeal joints, and metacarpophalangeal joints. Gottron's papules and cutaneous ulcers were also observed on the extensor surfaces of the joints. There was no joint tenderness, deformity, or lower limb edema. She exhibited muscle weakness with grade IV muscle strength and normal muscle tone on examination. Laboratory analyses indicated elevated inflammatory markers: erythrocyte sedimentation rate (ESR) 31 mm/H (range: 0–20 mm/H), serum ferritin (Ferr) 1,271.4 ng/ml (range: 11–306 ng/ml), and D dimer 1,186 ng/ml (range: <280 ng/ml). Additional laboratory tests revealed increased serum myogenic enzymes: alanine transaminase (ALT) at 345 U/L (range: 7–30 U/L), aspartate aminotransferase (AST) at 866 U/L (range: 14–44 U/L), lactic dehydrogenase (LDH) at 736 U/L (range: 165–395 U/L), and creatine kinase (CK) at 485 U/L (range: 40–200 U/L). The myositis spectrum was positive for anti-MDA5 and anti-RO-52 antibodies, while other autoantibodies and infection-related tests were negative. Electromyography indicated the presence of myogenic changes. Magnetic resonance imaging showed high-density areas in the femoral muscles on both sides, consistent with the characteristics of JDM. Chest CT imaging showed flocculent shadows in both lungs and local consolidation in the right lung (Figure 1A). The patient was diagnosed with anti-MDA5-positive JDM complicated by ILD and received combination therapy, including IVIG (2 g/kg/dose at a 1-month interval), glucocorticoid (2 mg/kg/day), oral hydroxychloroquine (5 mg/kg/day), tacrolimus (0.05 mg/kg/day), and tofacitinib (2.5 mg/dose, b.i.d.). The levels of myogenic enzymes, including ALT, AST, LDH, and CK, all decreased, with Ferr decreasing significantly from 1,271.4 to 316 ng/ml. Subsequent imaging showed improvement in pulmonary lesion absorption (Figure 1B). She also received trimethoprim-sulfadiazine as prophylactic treatment for opportunistic infections. However, her dyspnea progressed rapidly 20 days later, although without cough or sputum, with oxygen saturation dropping to 92%–93% at rest in room air, requiring high-flow nasal oxygen therapy. Serological tests for Epstein–Barr virus, cytomegalovirus, *Mycobacterium tuberculosis*, fungi, and mycoplasma were all negative. Her pulmonary status further deteriorated (Figure 1C), accompanied by elevated laboratory indicators (Ferr from 316 to 504.7 ng/ml, ALT from 335 to 350 U/L, and AST from 202 to 281 U/L). Given her diagnosis of RP-ILD, she was treated with intravenous cyclophosphamide (700 mg/m^2^), followed by three sessions of plasma exchange. This regimen provided symptomatic relief and improved laboratory indicators, but lung CT scans showed no significant changes (Figure 1D). Consequently, intravenous tocilizumab therapy was initiated (10 mg/kg) (Figure 2). The treatment was well tolerated, and subsequent lung CT scans revealed significant amelioration of bilateral pulmonary lesions after tocilizumab therapy (Figure 1E). The patient continued to receive oral glucocorticoid (2 mg/kg/day, with a gradual taper), hydroxychloroquine (5 mg/kg/day), tacrolimus (0.05 mg/kg/day), tofacitinib (2.5 mg/dose, b.i.d.), pirfenidone (50 mg/dose, t.i.d.), IVIG (2 g/kg/month, a total of six sessions), intravenous cyclophosphamide (500–700 mg/m^2^/month, a total of three sessions), and regular intravenous tocilizumab therapy (8–10 mg/kg/dose, once every 2 weeks for four times, with a gradual extension in the dosing interval to once monthly). Follow-up CT scans confirmed substantial improvement, showing significant absorption of lung inflammation and consolidation (Figure 1F). After 6 months of follow-up, the condition of the patient was stable, and the glucocorticoid dosage was gradually reduced to 5 mg/day.

## 3 Discussion and conclusion

We present the case of a Chinese girl diagnosed with anti-MDA5-positive JDM complicated by RP-ILD. The patient fulfills the diagnostic criteria set by Bohan and Peter in 1975 and the European Neuromuscular Centre (ENMC) in 2018. Notably, despite the absence of a muscle biopsy, this diagnosis has been conclusively reached. Initially, despite the administration of multiple immunosuppressive medications, the patient did not achieve remission. However, following treatment with tocilizumab, along with plasma exchange and other immunosuppressants, her disease activity, including myogenic enzymes, ferritin levels, and CT scans, quickly improved, allowing for a successful reduction in her glucocorticoid dosage.

JDM is the most common subtype of idiopathic inflammatory myopathy in children, with typical rashes (present in 100% of cases) and muscle weakness (in 90% of cases) being the most common manifestations at onset (1). The anti-MDA5 antibody, one of the myositis-specific autoantibodies first described by Sato et al. in 2005, is strongly associated with a high incidence of RP-ILD and lower survival rates. Reports indicate that the mortality rates for patients with anti-MDA5-positive dermatomyositis (DM) complicated by RP-ILD can be as high as 56%–80% (2, 3). The first 6 months, especially the first 3 months, are particularly high-risk periods, with more than 90% of RP-ILD cases occurring during this timeframe (2, 4). Therefore, all JDM patients, even those who are negative for anti-MDA5, should be closely monitored for the development of RP-ILD (4).

Treatment of RP-ILD in anti-MDA5-positive JDM is challenging. Given the rarity of this condition, current treatment recommendations are based on expert advice, as no official guidelines exist. The most common treatment strategy is triple therapy, which consists of high-dose glucocorticoids, calcineurin inhibitors, and intravenous cyclophosphamide. A prospective multicenter study conducted in Japan revealed that patients receiving triple therapy exhibited a significantly higher 6-month survival rate than those in the intensive control group (89% vs. 33%, respectively) (5). More recently, early combination therapy incorporating Janus-kinase (JAK) inhibitors has also yielded promising results. Chen et al. (6) showed that early initiation of JAK inhibitor-based combination therapy significantly improved the survival rate of patients with anti-MDA5-positive DM-ILD, with a cumulative 6-month survival rate of 100% in the tofacitinib group compared to 78% in the conventional immunosuppressant group. However, despite the combination of a JAK inhibitor with triple immunosuppressive therapy in our patient, the progression of lung lesions remained unchanged and continued to deteriorate.

Plasma exchange, which can remove antibodies and pro-inflammatory cytokines, has been reported as a rescue treatment option for intractable RP-ILD. In patients with anti-MDA5-positive DM, especially when the standard triple therapy alone is insufficient, adding plasma exchange to the regimen may enhance therapeutic effects and improve outcomes (7, 8). A study by Shirakashi et al. revealed that, among 13 patients with refractory anti-MDA5-positive DM-ILD, five of the eight patients who received plasma exchange survived, while all others who did not receive this treatment succumbed to the disease (8). In our patient, after three plasma exchange sessions, there was a significant decrease in ferritin and myogenic enzyme levels, yet the pulmonary CT scans showed no improvement. Pirfenidone has been added to conventional immunosuppressive treatment for DM-associated subacute interstitial pneumonia with pulmonary fibrosis. Data from a prospective study on antifibrotic agents indicated that mortality was lower in the group receiving pirfenidone compared to the control group (36.7% vs. 51.9%). Furthermore, in the subgroup with subacute ILD, the group receiving pirfenidone exhibited a significantly higher survival rate (90% vs. 44.4%, *P* = 0.045) (9). While antifibrotic therapies may offer ancillary benefits, at least in a subpopulation of MDA5-DM patients, the expert panel at the Arthritis and Rheumatism seminar may not recommend the use of pirfenidone in patients with RP-ILD associated with anti-MDA5 antibodies (10). Therefore, further studies will be required.

Biologics have been reported as a treatment option for patients with anti-MDA5-positive JDM complicated by RP-ILD. Rituximab is conditionally recommended as one of the first-line treatment options for RP-ILD (11). Case series have reported that four anti-MDA5-positive DM patients with RP-ILD showed improvement regarding respiratory symptoms, lung function tests, and chest imaging after rituximab infusions (12). There are also case reports/series of other biologics, such as infliximab, basiliximab, and daratumumab, but the number of cases is minimal, and larger prospective controlled studies are needed. Although the 2023 ACR guideline conditionally recommends against tocilizumab as a first-line treatment for RP-ILD in patients with systemic autoimmune rheumatic diseases (11), recent studies (13–15) have shown that the levels of inflammatory cytokines, such as type 1 interferon, interleukin (IL)-6, and IL-18, are significantly increased in patients with anti-MDA5-positive DM-ILD. IL-6, in particular, is closely related to the pathogenesis of this condition. A hyperinflammatory state is a distinctive feature of anti-MDA5-positive DM with RP-ILD. The serum IL-6 level in the RP-ILD subgroup of polymyositis (PM)/DM patients is higher than that in the non-ILD subgroup (16), suggesting that regulating this cytokine might offer a possible treatment strategy for RP-ILD. Only a few previous reports have indicated the use of tocilizumab for treating anti-MDA5-positive DM with RP-ILD. We searched the published literature in PubMed using keywords such as “MDA5,” “dermatomyositis,” “RP-ILD,” and “tocilizumab” (Table 1). As far as we know, all existing reports are limited to adult cases, with no studies involving children. This case is the first successful application of plasma exchange in combination with tocilizumab for treating RP-ILD in anti-MDA5-positive JDM patients. Among our case and the eight cases from the literature, no serious side effects have been reported.

**Table 1:** Published cases of tocilizumab treatment for RP-ILD associated with refractory anti-MDA5-positive DM.

	Gender	Age (years)	MSA/MAA	Clinical manifestations	Ferr (ng/ml)	Treatment	Dosage	Duration of TCZ treatment	Follow-up period	Outcome
Case 1 (17)	Male	43	Anti-MDA5	Rash, fever, dyspnea, dry cough, muscle weakness	1,825	GC, CYC, TAC, TCZ	480 mg/week	3 weeks	1 year	Improved
Case 2 (17)	Female	58	Anti-MDA5, anti-Ro-52	Rash, muscle weakness	471	GC, CYC, CsA, TCZ	NA	1 month	NA	Improved
Case 3 (17)	Male	64	Anti-MDA5	Rash, muscle weakness	1,145	GC, IVIG, CYC, TAC, TCZ	NA	6 months	NA	Improved
Case 4 (17)	Female	52	Anti-MDA5	Rash, swollen joints, weakness	463	GC, CYC, CsA, TCZ	NA	6 months	NA	Improved
Case 5 (17)	Male	42	Anti-MDA5, anti-Ro-52	Rash, fever	2,046	GC, IVIG, CYC/TAC, TCZ	NA		NA	Treatment withdrawn
Case 6 (17)	Female	50	Anti-MDA5, anti-Ro-52	Rash	1,329	GC, TAC/CYC, TCZ	NA	3 weeks	NA	Improved
Case 7 (18)	Male	31	Anti-MDA5, anti-Ro-52	Rash, fatigue, arthralgia, fever, dysphagia, hoarseness, cough	3,351	GC, IVIG, TAC, TCZ	480 mg/month	2 months	42 days	Improved
Case 8 (19)	Female	63	Anti-MDA5, anti-Ro-52	Dry cough, dyspnea	333	GC, IVIG, CYC, TAC, PE, TCZ	8 mg/kg week	3 weeks	2 months	Improved

anti-MDA5, anti-melanoma differentiation-associated gene 5; MSA, myositis-specific autoantibodies; MAA, myositis-associated autoantibodies; Ferr, ferritin; GC, glucocorticoid; IVIG, intravenous immunoglobulin; CYC, cyclophosphamide; TAC, tacrolimus; CsA, ciclosporin A; PE, plasma exchange; TCZ, tocilizumab; NA, not available.

In conclusion, tocilizumab proved to be effective and safe in our case, consistent with findings from previous reports. Our patient received six cycles of tocilizumab over 4 months. To the best of our knowledge, this is the first report demonstrating the efficacy of tocilizumab for treating anti-MDA5-positive JDM with RP-ILD. Anti-IL-6 therapy emerges as a novel approach for the treatment of refractory anti-MDA5-positive JDM with RP-ILD, particularly in cases where conventional treatments prove inadequate.

However, this experience is currently limited to a case report, and its findings may not be broadly generalizable. Some potential disease activity markers, such as IL-18 levels or anti-MDA5 antibody titers, were not systematically collected and serially monitored. Larger clinical studies and prospective trials are needed to accumulate more definitive evidence to validate these findings, particularly with respect to longer follow-up periods, the optimal duration and dosage of drug use, and potential adverse effects.

## Data Availability

The original contributions presented in the study are included in the article/Supplementary Material, further inquiries can be directed to the corresponding authors.
